# Supplementary material for: The ENCODE Uniform Analysis Pipelines
Source: bioRxiv. 2023 Apr 6:2023.04.04.535623. Preprint. [Version 1] doi: 10.1101/2023.04.04.535623 (PMC10104020; doi:10.1101/2023.04.04.535623)
Supplement: Supplement 1 [file media-1.pdf]

SamtoolsFlagstatsQualityMetric	DNase-seq, Total RNA-seq,
diff_chroms	flagstats: mate mapped different chr (mapQ>=5)
diff_chroms_qc_failed	flagstats: mate mapped different chr (mapQ>=5) - qc failed
duplicates	flagstats: duplicates
duplicates_qc_failed	flagstats: duplicates - qc failed
mapped	flagstats: mapped
mapped_pct	flagstats: mapped - percent
mapped_qc_failed	flagstats: mapped - qc failed
paired	flagstats: paired
paired_properly	flagstats: properly paired
paired_properly_pct	flagstats: properly paired - percent
paired_properly_qc_failed	flagstats: properly paired - qc failed
paired_qc_failed	flagstats: paired - qc failed
read1	flagstats: read1
read1_qc_failed	flagstats: read1 - qc failed
read2	flagstats: read2
read2_qc_failed	flagstats: read2 - qc failed
singletons	flagstats: singletons
singletons_pct	flagstats: singletons - percent
singletons_qc_failed	flagstats: singletons - qc failed
total	flagstats: total
total_qc_failed	flagstats: total - qc failed
with_itself	flagstats: with itself and mate mapped
with_itself_qc_failed	flagstats: with itself and mate mapped - qc failed
usable_fragments	Usable fragments, based on the mapped value.
**SamtoolsStatsQualityMetric**	**DNase-seq, WGBS**
1st fragments	samtools --stats: 1st fragments
average length	samtools --stats: average length
average quality	samtools --stats: average quality
bases duplicated	samtools --stats: bases duplicated
bases mapped	samtools --stats: bases mapped

bases mapped (cigar)	samtools --stats: bases mapped (cigar)
bases trimmed	samtools --stats: bases trimmed
error rate	samtools --stats: error rate
filtered sequences	samtools --stats: filtered sequences
insert size average	samtools --stats: insert size - average
insert size standard deviation	samtools --stats: insert size - standard deviation
inward oriented pairs	samtools --stats: inward oriented pairs
is sorted	samtools --stats: is sorted
last fragments	samtools --stats: last fragments
maximum length	samtools --stats: maximum length
mismatches	samtools --stats: mismatches
non-primary alignments	samtools --stats: non-primary alignments
outward oriented pairs	samtools --stats: outward oriented pairs
pairs on different chromosomes	samtools --stats: pairs on different chromosomes
pairs with other orientation	samtools --stats: pairs with other orientation
raw total sequences	samtools --stats: raw total sequences
reads MQ0	samtools --stats: reads MQ0
reads QC failed	samtools --stats: reads QC failed
reads duplicated	samtools --stats: reads duplicated
reads mapped	samtools --stats: reads mapped
reads mapped and paired	samtools --stats: reads mapped and paired
reads paired	samtools --stats: reads paired
reads properly paired	samtools --stats: reads properly paired
reads unmapped	samtools --stats: reads unmapped
sequences	samtools --stats: sequences
total length	samtools --stats: total length
**CorrelationQualityMetric**	**Deprecated**
Pearson correlation	Pearson's R correlation
Spearman correlation	Spearman's rank correlation
Items	Count of items from two different datasets that are being correlated

Standard deviation	Standard deviation of difference
MAD of log ratios	Mean-Average-Deviation (MAD) of replicate log ratios from quantification
Details	Description of methods
