# Supplementary material for: The ENCODE Uniform Analysis Pipelines
Source: bioRxiv. 2023 Apr 6:2023.04.04.535623. Preprint. [Version 1] doi: 10.1101/2023.04.04.535623 (PMC10104020; doi:10.1101/2023.04.04.535623)
Supplement: Supplement 2 [file media-2.pdf]

HicQualityMetric	
sequenced_reads	Number of total reads sequenced for library
sequenced_read_pairs	Number of total read pairs sequenced for library
no_chimera_found	Number of reads where no chimera was found
0_alignments	Number of reads that did not align to the reference genome
1_alignment	Number of reads that align to one position in the reference genome
1_alignment_unique	Number of unique reads that align to one position in the reference genome
1_alignment_duplicates	Number of duplicate reads that align to one position in the reference genome
2_alignments	Number of reads that align to two positions in the reference genome
2_alignments_a_b	Number of reads that align to two positions in the reference genome of the type A...B
2_alignment_duplicates	Number of duplicate reads that align to two positions in the reference genome
2_alignment_unique	Number of unique reads that align to two positions in the reference genome
2_alignments_a1_a2b_a1b2_b1a2	Number of reads that align to two positions in the reference genome of the type A1...A2B; A1B2...B1A2
3_or_more_alignments	Number of reads that align to three or more positions in the reference genome
pct_no_chimera_found	Percent of reads where no chimera was found
pct_0_alignments	Percent of reads that did not align to the reference genome
pct_1_alignment	Percent of reads that align to one position in the reference genome
pct_1_alignment_unique	Percent of unique reads that align to one position in the reference genome
pct_1_alignment_duplicates	Percent of duplicate reads that align to one position in the reference genome
pct_sequenced_1_alignment_unique	Percent of unique reads that align to one position in the reference genome
pct_sequenced_1_alignment_duplicates	Percent of duplicate reads that align to one position in the reference genome
pct_2_alignments	Percent of reads that align to two positions in the reference genome
pct_2_alignment_unique	Percent of unique reads that align to two positions in the reference genome
pct_2_alignment_duplicates	Percent of duplicate reads that align to two positions in the reference genome
pct_2_alignments_a_b	Percent of reads that align to two positions in the reference genome of the type A...B
pct_2_alignments_a1_a2b_a1b2_b1a2	Percent of reads that align to two positions in the reference genome of the type A1...A2B; A1B2...B1A2
pct_sequenced_2_alignment_unique	Percent of unique reads that align to two positions in the reference genome
pct_sequenced_2_alignment_duplicates	Percent of duplicate reads that align to two positions in the reference genome
pct_3_or_more_alignments	Percent of reads that align to three or more positions in the reference genome
pct_unique_total_duplicates	Percent of duplicate reads that align to the reference genome
pct_unique_total_unique	Percent of unique reads that align to the reference genome
one_or_both_reads_unmapped	Number of read pairs where one or both reads is unmapped

pct_one_or_both_reads_unmapped	Percent of read pairs where one or both reads is unmapped
ligation_motif_present	Number of reads containing a ligation motif
pct_ligation_motif_present	Percent of reads containing a ligation motif
avg_insert_size	Average insert size
total_unique	Number of unique reads that align to the reference genome
pct_sequenced_total_unique	Percent of unique reads that align to the reference genome
total_duplicates	Number of duplicate reads that align to the reference genome
pct_sequenced_total_duplicates	Percent of duplicate reads that align to the reference genome
library_complexity_estimate	Estimate of Library Complexity
library_complexity_estimate_1_alignment	Estimate of Library Complexity (for reads with one alignment)
library_complexity_estimate_2_alignments	Estimate of Library Complexity (for reads with two alignments)
library_complexity_estimate_1_and_2_alignments	Estimate of Library Complexity (for reads with one and two alignments)
intra_fragment_reads	Number of intra-fragment reads
pct_sequenced_intra_fragment_reads	Percent of intra-fragment reads
pct_unique_intra_fragment_reads	Percent of unique intra-fragment reads
below_mapq_threshold	Number of reads below the mapQ threshold
pct_sequenced_below_mapq_threshold	Percent of reads below the mapQ threshold
pct_unique_below_mapq_threshold	Percent of unique reads below the mapQ threshold
hic_contacts	Number of Hi-C contacts
pct_sequenced_hic_contacts	Percent of Hi-C contacts
pct_unique_hic_contacts	Percent of unique Hi-C contacts
pct_5_prime_bias_long_range	Percent of read ends from long range contacts mapping closer to the 5’ end of the fragment
pct_3_prime_bias_long_range	Percent of read ends from long range contacts mapping closer to the 3’ end of the fragment
lior_convergence	Distance in basepairs at which left, right, inner, and outer pair types converge
pct_left_pair_type	Percentage of Hi-C contacts with left pair type
pct_right_pair_type	Percentage of Hi-C contacts with right pair type
pct_inner_pair_type	Percentage of Hi-C contacts with inner pair type
pct_outer_pair_type	Percentage of Hi-C contacts with outer pair type
inter_chromosomal	Number of inter-chromosomal Hi-C contacts
pct_sequenced_inter_chromosomal	Percent of inter-chromosomal Hi-C contacts
pct_unique_inter_chromosomal	Percent of unique inter-chromosomal Hi-C contacts
intra_chromosomal	Number of intra-chromosomal Hi-C contacts

pct_sequenced_intra_chromosomal	Percent of intra-chromosomal Hi-C contacts
pct_unique_intra_chromosomal	Percent of unique intra-chromosomal Hi-C contacts
short_range_less_than_500bp	Number of intra-chromosomal Hi-C contacts with loci less than 500 basepairs apart
pct_sequenced_short_range_less_than_500bp	Percent of intra-chromosomal Hi-C contacts with loci less than 500 basepairs apart
pct_unique_short_range_less_than_500bp	Percent of unique intra-chromosomal Hi-C contacts with loci less than 500 basepairs apart
short_range_500bp_to_5kb	Number of intra-chromosomal Hi-C contacts with loci between 500 basepairs and 5 kilobases apart
pct_sequenced_short_range_500bp_to_5kb	Percent of intra-chromosomal Hi-C contacts with loci between 500 basepairs and 5 kilobases apart
pct_unique_short_range_500bp_to_5kb	Percent of unique intra-chromosomal Hi-C contacts with loci between 500 basepairs and 5 kilobases apart
short_range_5kb_to_20kb	Number of intra-chromosomal Hi-C contacts with loci between 5 and 20 kilobases apart
pct_sequenced_short_range_5kb_to_20kb	Percent of intra-chromosomal Hi-C contacts with loci between 5 and 20 kilobases apart
pct_unique_short_range_5kb_to_20kb	Percent of unique intra-chromosomal Hi-C contacts with loci between 5 and 20 kilobases apart
long_range_greater_than_20kb	Number of intra-chromosomal Hi-C contacts with loci greater than 20 kilobases apart
pct_sequenced_long_range_greater_than_20kb	Percent of intra-chromosomal Hi-C contacts with loci greater than 20 kilobases apart
pct_unique_long_range_greater_than_20kb	Percent of unique intra-chromosomal Hi-C contacts with loci greater than 20 kilobases apart
