# Supplementary material for: The ENCODE Uniform Analysis Pipelines
Source: bioRxiv. 2023 Apr 6:2023.04.04.535623. Preprint. [Version 1] doi: 10.1101/2023.04.04.535623 (PMC10104020; doi:10.1101/2023.04.04.535623)
Supplement: Supplement 3 [file media-3.pdf]

StarQualityMetric	total RNA-seq, microRNA-seq
% of chimeric reads	STAR % of chimeric reads
% of reads mapped to multiple loci	STAR % of reads mapped to multiple loci
% of reads mapped to too many loci	STAR % of reads mapped to too many loci
% of reads unmapped: other	STAR % of reads unmapped: other
% of reads unmapped: too many mismatches	STAR % of reads unmapped: too many mismatches
% of reads unmapped: too short	STAR % of reads unmapped: too short
Average input read length	STAR Average input read length
Average mapped length	STAR Average mapped length
Deletion average length	STAR Deletion average length
Deletion rate per base	STAR Deletion rate per base
Insertion average length	STAR Insertion average length
Insertion rate per base	STAR Insertion rate per base
Mapping speed, Million of reads per hour	STAR Mapping speed, Million of reads per hour
Mismatch rate per base, %	STAR Mismatch rate per base, %
Number of chimeric reads	STAR Number of chimeric reads
Number of input reads	STAR Number of input reads
Number of reads mapped to multiple loci	STAR Number of reads mapped to multiple loci
Number of reads mapped to too many loci	STAR Number of reads mapped to too many loci
Number of splices: AT/AC	STAR Number of splices: AT/AC
Number of splices: Annotated (sjdb)	STAR Number of splices: Annotated (sjdb)
Number of splices: GC/AG	STAR Number of splices: GC/AG
Number of splices: GT/AG	STAR Number of splices: GT/AG
Number of splices: Non-canonical	STAR Number of splices: Non-canonical
Number of splices: Total	STAR Number of splices: Total
Uniquely mapped reads %	STAR Uniquely mapped reads %
Uniquely mapped reads number	STAR Uniquely mapped reads number
read_depth	Sum of the uniquely mapped reads number and the number of reads mapped to multiple loci.

GeneQuantificationQualityMetric	total RNA-seq
number_of_genes_detected	Number of Genes Detected

GeneTypeQuantificationQualityMetric	total RNA-seq
Mt_rRNA	Number of reads assigned to transcripts from the \"Mt_rRNA\" GENCODE biotype; mitochondrial rRNAs
antisense	Number of reads in transcripts that overlap the genomic span (i.e. exon or introns) of a protein-coding locus on the opposite strand.
miRNA	Number of reads assigned to transcripts from the \"miRNA\" GENCODE biotype; microRNAs

processed_transcript	Number of reads mapped to genomic regions which don't contain an ORF.
protein_coding	Number of reads assigned to transcripts from the \"protein_coding\" GENCODE biotype; contain ORFs
rRNA	Number of reads assigned to transcripts from the \"rRNA\" GENCODE biotype; encode for rRNAs
ribozyme	Number of reads assigned to transcripts from the \"ribozyme\" GENCODE biotype; encode for ribozymes
sRNA	Number of reads assigned to transcripts from the \"sRNA\" GENCODE biotype; encode for sRNAs
scaRNA	Number of reads assigned to transcripts from the \"scaRNA\" GENCODE biotype; encode for scaRNAs
sense_intronic	Number of reads in long non-coding transcript in introns of a coding gene that does not overlap any exons.
sense_overlapping	Number of reads in long non-coding transcript that contains a coding gene in its intron on the same strand.
snRNA	Number of reads assigned to transcripts from the \"snRNA\" GENCODE biotype; encode for snRNAs
snoRNA	Number of reads assigned to transcripts from the \"snoRNA\" GENCODE biotype; encode for snoRNAs
spikein	Number of reads assigned to transcripts from the spike ins
**MadQualityMetric**	**total RNA-seq**
SD of log ratios	Standard Deviation of replicate log ratios from quantification
Pearson correlation	Pearson correlation coefficient of replicates from quantification
Spearman correlation	Spearman correlation coefficient of replicates from quantification
MAD of log ratios	Mean-Average-Deviation (MAD) of replicate log ratios from quantification
**MicroRnaMappingQualityMetric**	**microRNA-seq**
aligned_reads	Number of aligned reads
**MicroRnaQuantificationQualityMetric**	**microRNA-seq**
expressed_mirnas	Number of miRNAs expressed
**LongReadRnaMappingQualityMetric**	**long read RNA-seq**
full_length_non_chimeric_read_count	Quantity of reads that are full-length and do not contain a chimeric adaptor arrangement.
mapping_rate	Proportion of reads mapping to the genome
**LongReadRnaQuantificationQualityMetric**	**long read RNA-seq**
genes_detected	Number of GENCODE genes detected for the replicate
