# Supplementary material for: The ENCODE Uniform Analysis Pipelines
Source: bioRxiv. 2023 Apr 6:2023.04.04.535623. Preprint. [Version 1] doi: 10.1101/2023.04.04.535623 (PMC10104020; doi:10.1101/2023.04.04.535623)
Supplement: Supplement 4 [file media-4.pdf]

**DnaseAlignmentQualityMetric**	
fourier_transform_eleven	Fourier transform of the insert size at 11 bases
insert_size_histogram	Insert size histogram for all reads
insert_size_metric	Insert size metric file
large_small_ratio	Ratio of long to short reads
nuclear_preseq	Total read metrics file
nuclear_preseq_targets	Sequencing depth file
**DnaseFootprintingQualityMetric**	
footprint_count	Total number of DNaseI footprints
**DuplicatesQualityMetric**	
Reads Examined	Total number of paired and unpaired reads examined
Read Duplicates	Total number of paired and unpaired read duplicates
UMI Read Duplicates	Number of UMI flagged read duplicates
Percent Duplication	Percent of reads that are duplicates
Read Pairs Examined	Number of read pairs examined by picard
Read Pair Duplicates	Number of read pairs detected as duplicates by picard
Read Pair Optical Duplicates	Number of read pairs detected as optical duplicates by picard
Unmapped Reads	Number of unmapped detected by picard
Unpaired Reads Examined	Number of unpaired reads examined by picard
Unpaired Read Duplicates	Number of unpaired reads detected as duplicates by picard
Estimated Library Size	Library size in reads as estimated by picard
**HotspotQualityMetric**	
spot1_score	SPOT score as calculated by Hotspot1
spot2_score	SPOT score as calculated by Hotspot2
hotspot_count	Count of hotspots discovered by Hotspot2
peaks_count	Count of peaks discovered by Hotspot2
total_tags	Count of read tags provided to Hotspot
hotspot_tags	Count of read tags discovered to be in hotspots
five_percent_allcalls_count	Count of five percent calls

five_percent_hotspots_count	Count of five percent hotspots
five_percent_narrowpeaks_count	Count of five percent narrowpeaks
tenth_of_one_percent_narrowpeaks	Count of tenth of one percent narrowpeaks
**TrimmingQualityMetric**	
PE read-pairs processed	Total number read-pairs processed
PE read-pairs trimmed	Total number read-pairs trimmed
read_1_with_adapter	Number of read1 with adapter
read_2_with_adapter	Number of read2 with adapter
SE reads processed	Total number (single-end) reads processed
SE reads trimmed	Total number (single-end) reads trimmed
total_read_pairs_processed	Total number read-pairs processed
**EdwbamstatsQualityMetric**	**Deprecated**
alignedBy	The aligner used
isPaired	If alignment is from paired-end reads this will be set to 1
isSortedByTarget	If the bam is sorted by target location, this is set to 1; if sorted by name, this is set to 0
mappedCount	Count of mapped reads
readBaseCount	Count of total bases in reads
readCount	Count of all reads
readSizeMax	Longest read
readSizeMean	Mean size of all reads
readSizeMin	Shortest read
readSizeStd	Standard deviation of read size
targetBaseCount	Number of bases covered in target (e.g. on chromosomes)
targetSeqCount	Count of target sequences (e.g. chromosomes)
u4mReadCount	Number of randomly sampled uniquely mapped items used in complexity calculation
u4mUniquePos	Number of unique positions that sampled uniquely mapped reads were mapped to
u4mUniqueRatio	Ratio of unique positions to uniquely mapped reads
uniqueMappedCount	Count of reads uniquely mapped

FilteringQualityMetric	Deprecated
pre-filter all reads	Count of all reads prior to filtering
pre-filter mapped reads	Count of mapped reads prior to filtering
post-filter all reads	Count of all reads after filtering
post-filter mapped reads	Count of mapped reads after filtering
