# Supplementary material for: The ENCODE Uniform Analysis Pipelines
Source: bioRxiv. 2023 Apr 6:2023.04.04.535623. Preprint. [Version 1] doi: 10.1101/2023.04.04.535623 (PMC10104020; doi:10.1101/2023.04.04.535623)
Supplement: Supplement 5 [file media-5.pdf]

CpgCorrelationQualityMetric	
CpG pairs	Number of CpG pairs
CpG pairs with atleast 10 reads each	CpG pairs with atleast 10 reads each
Pearson correlation	Pearson's correlation of CpG pairs with at least 10 reads each
**GembsAlignmentQualityMetric**	
sequenced_reads	Number of sequenced reads
unmapped_reads	Number of unmapped reads
pct_unmapped_reads	Percentage of unmapped reads
correct_pairs	Number of correct pairs
general_reads	Number of general reads
average_coverage	Average coverage
pct_general_reads	Percentage of general reads
unique_fragments	Number of unique fragments
pct_unique_fragments	Percentage of unique fragments
conversion_rate	Bisulfite conversion rate
reads_under_conversion_control	Number of reads under conversion control
pct_reads_under_conversion_control	Percentage of reads under conversion control
reads_over_conversion_control	Number of reads over conversion control
pct_reads_over_conversion_control	Percentage of reads over conversion control
bisulfite_reads_c2t	Number of bisulfite reads C2T
pct_bisulfite_reads_c2t	Percentage of bisulfite reads C2T
bisulfite_reads_g2a	Number of bisulfite reads G2A
pct_bisulfite_reads_g2a	Percentage of bisulfite reads G2A
