# Supplementary material for: The ENCODE Uniform Analysis Pipelines
Source: bioRxiv. 2023 Apr 6:2023.04.04.535623. Preprint. [Version 1] doi: 10.1101/2023.04.04.535623 (PMC10104020; doi:10.1101/2023.04.04.535623)
Supplement: Supplement 7 [file media-7.pdf]

**AtacAlignmentEnrichmentQualityMetric**	
subsampled_reads	Number of reads subsampled from the sample for calculation of all cross-correlation metrics
fri_dhs	Fraction of reads in universal DHS regions
fri_exclusion_list	Fraction of reads in regions from exclusion list
fri_prom	Fraction of reads in promoter regions
fri_enh	Fraction of reads in enhancer regions
estimated_fragment_len	Fragment length/strandshift. This is the estimated fragment length/strand shift for each dataset as estimated by strand shift cross-correlation analysis
corr_estimated_fragment_len	Value of correlation function at estimated fragment length.
phantom_peak	Strand shift value at which the phantom (false) peak in cross-correlation is observed. This is typically the read length.
corr_phantom_peak	Value of cross-correlation function at strand shift corresponding to phantom_peak (which is typically the read length).
argmin_corr	Strand shift corresponding to the minimum value of cross-correlation (min_corr).
min_corr	Minimum cross-correlation over a sufficiently wide range of strand shifts (typically -100 bp to ~3 times the expected size of DNA fragments based on sonication and size selection protocols).
NSC	Normalized strand cross-correlation = FRAGLEN_CC / MIN_CC. Ratio of strand cross-correlation at estimated fragment length to the minimum cross-correlation over all shifts.
RSC	Relative cross-correlation coefficient. Ratio of strand cross-correlation at fragment length and at read length
auc	The “area under the curve”, with a maximum value of 0.5. Lower values generally indicate higher and more focal enrichment.
syn_auc	The expected area under the curve of a perfectly behaved input sample having the same mean sequencing depth of a given sample. This is useful to put the observed AUC into perspective.
x_intercept	The point (on the X-axis) at which the curve is 0. This is approximately the percentage of the genome that was not sequenced in a particular sample.
syn_x_intercept	The expected X-intercept of a perfectly behaved input sample having the same mean sequencing depth of a given sample. This is useful to put the observed X-intercept into perspective.
elbow_pt	The elbow point attempts to measure the position at which the line turns upward. In practice, this is the point at which the plotted line is furthest from the line from the lower-left to the upper-right corner of the graph (equivalent to a perfect input sample with infinite coverage). The point returned is the position on the X-axis of this elbow point and higher values indicate more enrichment.
syn_elbow_pt	The expected elbow point of a perfectly behaved input sample having the same mean sequencing depth of a given sample. This is useful to put the observed elbow point into perspective.
jsd	The Jensen-Shannon distance between the replicate and the control.
syn_jsd	The Jensen-Shannon distance between a given sample and a perfect input sample with the same coverage depth (i.e., the plot generated from the Poisson probability mass function with lambda equal to the mean coverage in the sample).
pct_genome_enrich	The approximate percentage of the genome enriched in signal (e.g., bound by a transcription factor or having a certain histone modification).
diff_enrich	The differential enrichment between a given sample and that indicated by --JSDsample at the elbow point.
ch_div	The CHANCE divergence between the replicate and the control.
tss_enrichment	Enrichment metric. Fold change of average ATAC-seq signal coverage at gene TSSs relative to background bins distal (typically 2Kb away) from TSS.
**AtacAlignmentQualityMetric**	
total_reads	Number of total reads passing QC"
total_reads_qc_failed	Number of total reads failing QC
duplicate_reads	Number of reads with duplicates passing QC
duplicate_reads_qc_failed	Number of reads with duplicates failing QC
mapped_reads	Number of mapped reads passing QC
mapped_reads_qc_failed	Number of mapped reads failing QC
pct_mapped_reads	Percent of mapped reads passing QC
paired_reads	Number of paired reads passing QC
paired_reads_qc_failed	Number of paired reads failing QC
read1	Number of read1 reads passing QC
read1_qc_failed	Number of read1 reads failing QC
read2	Number of read2 reads passing QC
read2_qc_failed	Number of read2 reads failing QC
properly_paired_reads	Number of properly paired reads passing QC
properly_paired_reads_qc_failed	Number of properly paired reads failing QC
pct_properly_paired_reads	Percent of properly paired reads passing QC
with_itself	Number of reads with both itself & mate mapped passing QC
with_itself_qc_failed	Number of reads with both itself & mate mapped failing QC
singletons	Number of singletons (unpaired reads) passing QC
singletons_qc_failed	Number of singletons (unpaired reads) failing QC
pct_singletons	Percent of singletons (unpaired reads) passing QC
diff_chroms	Number of reads with mate mapped to different chromosomes passing QC
diff_chroms_qc_failed	Number of reads with mate mapped to different chromosomes failing QC
non_mito_reads	Number of non-mitochondrial reads
mito_reads	Number of mitochondrial reads
frac_mito_reads	Fraction of mitochondrial reads
frac_reads_in_nfr	Fraction of reads in nucleosome free region (NFR)
frac_reads_in_nfr_qc_pass	Whether NFR/mono-nuc reads passes QC
frac_reads_in_nfr_qc_reason	Reason for frac_reads_in_nfr_qc_pass
nfr_over_mono_nuc_reads	Ratio of reads in nucleosome free region (NFR) to reads in mononucleosome region (Mono-Nuc).
nfr_over_mono_nuc_reads_qc_pass	Whether NFR/mono-nuc reads passes QC
nfr_over_mono_nuc_reads_qc_reason	Reason for nfr_over_mono_nuc_reads_qc_pass
nfr_peak_exists	Whether NFR peak exists
mono_nuc_peak_exists	Whether mononucleosome peak exists
di_nuc_peak_exists	Whether there are peaks in dinucleosome regions (Di-Nuc).
usable_fragments	Usable fragments, based on the mapped_reads value.
**AtacLibraryQualityMetric**	
unpaired_reads	Number of unpaired reads before filtering
paired_reads	Number of paired reads before duplicate filtering
unmapped_reads	Number of unmapped reads before duplicate filtering
unpaired_duplicate_reads	Number of unpaired duplicates before duplicate filtering
paired_duplicate_reads	Number of paired duplicates before duplicate filtering
paired_optical_duplicate_reads	Number of paired optical duplicates before duplicate filtering
pct_duplicate_reads	Percent of paired duplicates before duplicate filtering
total_fragments	Number of fragments before duplicate filtering
distinct_fragments	Number of distinct fragments
positions_with_one_read	Number of locations to which exactly one read (pair) maps
NRF	Non redundant fraction (indicates library complexity). Number of distinct unique mapping reads (i.e. after removing duplicates) / Total number of reads
PBC1	PCR Bottlenecking coefficient 1 = M1/M_DISTINCT where M1: number of genomic locations where exactly one read maps uniquely, M_DISTINCT: number of distinct genomic locations to which some read maps uniquely
PBC2	PCR Bottlenecking coefficient 2 (indicates library complexity) = M1/M2 where M1: number of genomic locations where only one read maps uniquely and M2: number of genomic locations where 2 reads map uniquely
**AtacPeakEnrichmentQualityMetric**	
frip	Fraction of reads in the peak file
min_size	Smallest peak width
25_pct	25th percentile of peak widths
50_pct	50th percentile of peak widths
75_pct	75th percentile of peak widths
max_size	Largest peak width
mean	Mean peak width

**AtacReplicationQualityMetric**	
reproducible_peaks	Number of peaks called from one replicate or pooled replicates passing reproducibility test by comparing self-pseudoreplicates or pooled pseudo-replicates.
idr_cutoff	Irreproducible Discovery Rate (IDR) threshold used to define reproducible peaks
rescue_ratio	max(Np,Nt) / min (Np,Nt); Np: Pooled-pseudoreplicate consistent peaks (comparing two pseudoreplicates generated by subsampling pooled reads from Rep1 and Rep2); Nt: True Replicate consistent peaks (comparing true replicates Rep1 vs Rep2)
self_consistency_ratio	max(N1,N2) / min (N1,N2); N1: Replicate 1 self-consistent peaks (comparing two pseudoreplicates generated by subsampling Rep1 reads); N2: same as N1 for Rep2
reproducibility	Reproducibility test result for this experiment (pass/fail)
